# Sodium Chloride Nanoparticles Potentiate Radiation Therapy by Disrupting Osmolarity Balance and Enhancing Antitumor Immunity

**DOI:** 10.1021/acs.nanolett.5c03022

**Published:** 2025-09-15

**Authors:** Shuyue Zhan, Jianwen Li, Xinning Lai, Yaochao Zheng, Zhizi Feng, Sahil Bakul Patel, Wei Yang, Yong Teng, Yao Yao, Jin Xie

**Affiliations:** † Department of Chemistry, 1355 University of Georgia, Athens, Georgia 30602, United States; ‡ Regenerative Bioscience Center, Department of Animal and Dairy Science, 1355 University of Georgia, Athens, Georgia 30602, United States; § Department of Hematology and Medical Oncology & Winship Cancer Institute, 1371 Emory University School of Medicine, Atlanta, Georgia 30322, United States; ∥ Department of Pharmaceutical & Biomedical Sciences, 1355 University of Georgia, Athens, Georgia 30602, United States; ⊥ School of Chemical, Materials and Biomedical Engineering, College of Engineering, The University of Georgia, Athens, Georgia 30602, United States

**Keywords:** Nanoparticles, radiosensitizer, osmolarity, sodium/calcium exchanger, antitumor immunity, radiation

## Abstract

This study investigates sodium chloride nanoparticles (SCNPs) as radiosensitizers. In contrast to conventional radiosensitizers that rely on high-Z effects or DNA-targeted mechanisms, SCNPs potentiate radiation-induced cellular damage by perturbing ion homeostasis. Importantly, SCNPs by elevating intracellular sodium levels reverse the sodium/calcium exchanger (NCX), leading to calcium influx. This calcium surge not only amplifies radiation-induced cancer cell death but also activates the cGAS-STING pathway, leading to the production of type I interferons. In syngeneic head and neck cancer models, SCNPs significantly improve tumor control and long-term survival in combination with radiation, without inducing detectable toxicity. Mechanistic studies reveal that these therapeutic benefits are largely immune-mediated, demonstrated by enhanced dendritic cell maturation and increased tumor infiltration of T cells. Overall, SCNPs are poised to overcome the limitations of conventional radiosensitizers, such as systemic toxicity and reduced efficacy with megavoltage beams, and offer a mechanistically distinct approach with significant translational potential.

## Graphical abstract

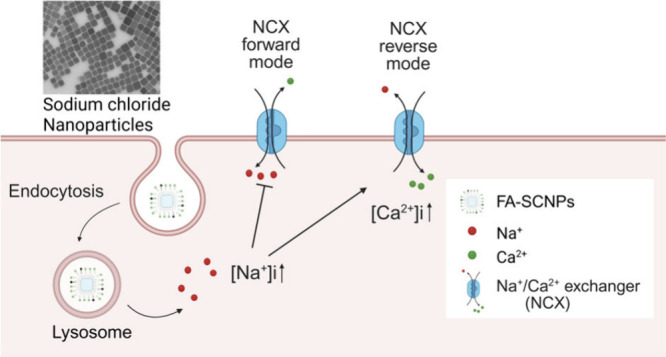

Radiosensitizers are agents administered during radiation therapy to enhance the killing of cancer cells.^1^ Despite advances in radiation delivery, unintended damage to healthy tissue remains inevitable, limiting the maximum radiation dose that can be safely administered. Radiosensitizers can be added to improve locoregional tumor control and reduce the risk of metastasis within the dose limits. Since DNA has traditionally been considered the primary biological target of radiotherapy, most current radiosensitizers are chemotherapeutics intended to enhance radiation-induced DNA damage, acting by directly increasing DNA damage, arresting cancer cells in the radiosensitive G2/M phase, or inhibiting DNA repair mechanisms.^2^ A major limitation of this combined chemo-radiotherapy approach is that it is often associated with severe systemic toxicity, leaving many patients unable to tolerate or complete a full course of treatment.^3^

The development of nanomaterials has provided emerging opportunities to enhance the efficacy of radiotherapy. Among them, nanoparticles composed of high atomic number (Z) materials have been extensively investigated as radiosensitizers.^4,5^ Due to their large cross sections for high-energy beams and enhanced photoelectric effects, high-Z nanoparticles, when localized in tumors, can increase the production of secondary electrons and reactive oxygen species (ROS), thereby amplifying radiation-induced damage at the same physical dose. Notable examples include NBTXR3, an intratumoral formulation composed primarily of hafnium oxide nanoparticles, which has been approved in the European Union for the treatment of soft tissue sarcoma and is currently undergoing clinical trials in the United States for various malignancies, including head and neck cancer.^6,7^ Other high-Z nanoparticles such as gold, silver, gadolinium and iron oxide have also been evaluated in preclinical studies as intratumoral radiosensitizers.^8–11^ While these advances are promising, concerns remain regarding the potential toxicity and biological clearance of high-Z nanoparticles. In addition, although the photoelectric effect is pronounced with the kilovoltage radiation commonly used in preclinical studies, its efficacy is significantly reduced with the megavoltage beams used in clinical radiotherapy.^12,13^ These challenges have hindered the clinical translation of high-Z nanoparticle-based radiosensitizers.

In this study, we investigate sodium chloride nanoparticles (SCNPs) as a class of radiosensitizers that function independently of the high-Z effect. Our previous work demonstrated that surface-modified SCNPs can deliver sodium and chloride ions into cancer cells, in doing so disrupting osmolarity and redox homeostasis.^14^ Here, we show that SCNPs, when combined with radiation, significantly amplify oxidative stress in cancer cells, thereby enhancing radiation-induced cell death in head and neck cancer models. Interestingly, we found that this radiosensitizing effect is mediated, at least in part, by calcium influx through the sodium/calcium exchanger (NCX), resulting in intracellular calcium overload that is detrimental to cell survival. In addition, we show that SCNPs enhance the radiation-induced immune response, evidenced by increased dendritic cell (DC) maturation and antigen cross-presentation, leading to enhanced T cell tumor infiltration. This stimulatory effect is also linked to calcium influx, which enhances radiation-induced activation of the cGAS-STING pathway, causing increased secretion of type I interferons. Importantly, SCNPs degrade to saline after treatment, which is safely absorbed by the host, avoiding systemic toxicity commonly associated with chemotherapeutic agents or high Z nanoparticle radiosensitizers. Overall, this work introduces a distinct nanoparticle-based radiosensitization strategy that enhances both local tumor control and systemic immune responses, offering a promising alternative to current radiosensitizer approaches.

We synthesized SCNPs using a nonaqueous coprecipitation method. Briefly, sodium acetate was dissolved in ethanol, and acetyl chloride in hexane was added dropwise to this mixture, along with oleylamine as a surfactant (Figure 1a). The nanoparticle products were collected by centrifugation. Transmission electron microscopy revealed that the resulting nanoparticles measured 284.8 ± 48.0 nm in size (Figure 1b). Energy-dispersive X-ray spectroscopy (EDS) and elemental mapping confirmed the nanoparticles were composed of NaCl (Figure 1c,d). The nanoparticles were then coated with a phospholipid layer consisting of DSPE-PEG(2000) Amine and DSPE-PEG(2000) Folate at a 9:1 molar ratio. Folate was included as a targeting ligand considering that folate receptor is overexpressed in many types of cancer, including head and neck cancer.^15,16^ The resulting folate-functionalized SCNPs (FA-SCNPs) (Figure 1a) exhibited a hydrodynamic diameter of 256.1 ± 15.3 nm and a slightly negative surface charge (Figure 1e,f).

**Figure 1:**
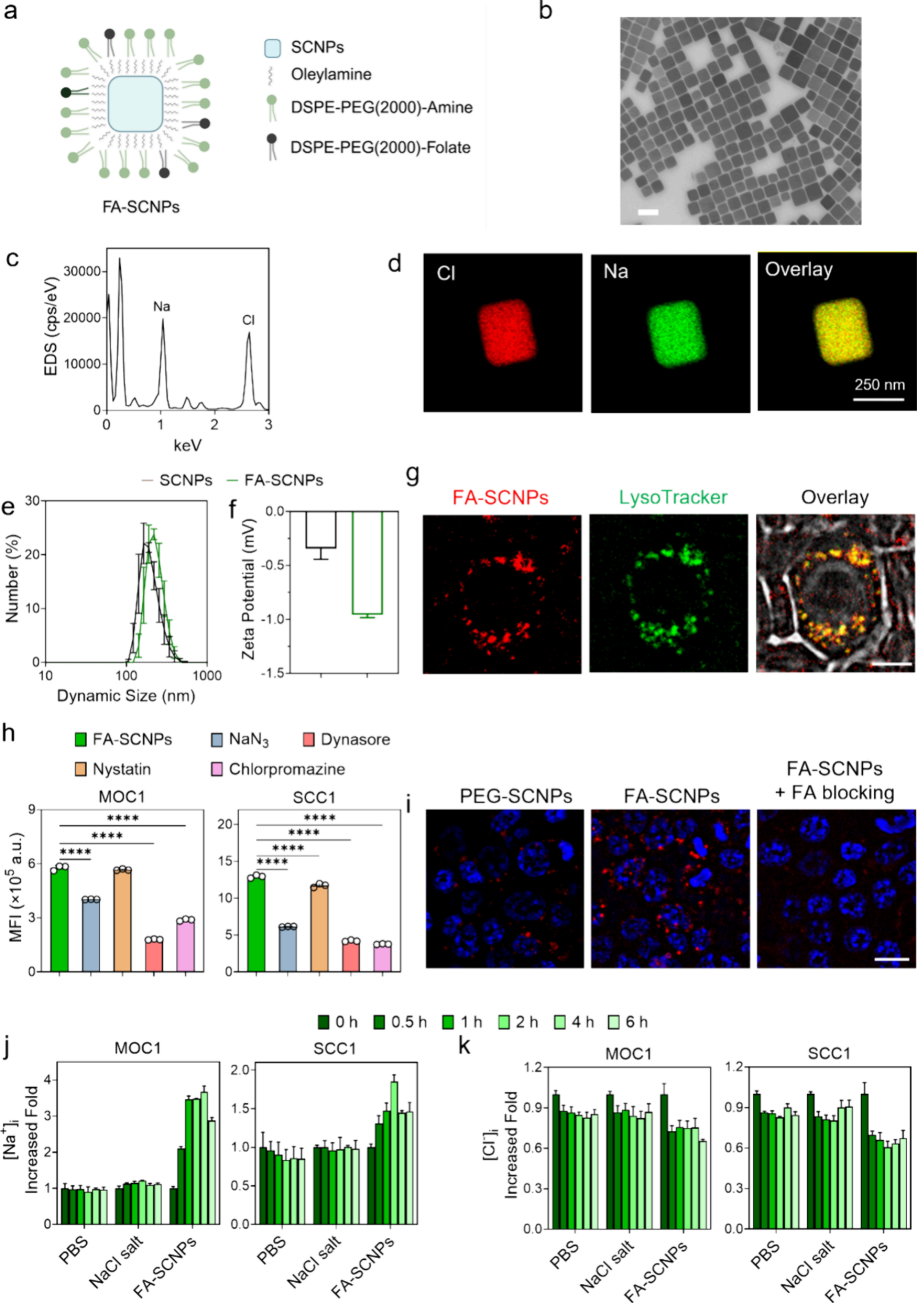
Synthesis and cellular uptake of SCNPs. (a) Schematic illustration of the composition of FA-SCNPs. (b) Representative TEM image of as-synthesized SCNPs. Scale bar, 500 nm. (c) EDS spectrum of SCNPs. (d) TEM image of SCNP with elemental mapping by EDS. (e) DLS analysis of SCNPs and FA-SCNPs in water. (f) Zeta potential of SCNPs and FA-SCNPs, measured in PBS. (g) Cellular uptake of Rhodamine B (RB)-labeled FA-SCNPs by MOC1 cells, visualized by fluorescence microscopy. Lysosomes were stained with LysoTracker. Scale bar: 20 μm. (h) Cellular uptake of RB-labeled FA-SCNPs in the presence of endocytosis inhibitors, including NaN_3_, dynasore, nystatin, and chlorpromazine, assessed by flow cytometry. Studies were conducted on both MOC1 and SCC1 cells. (i) Microscopic images comparing the uptake of RB-labeled SCNPs or FA-SCNPs by MOC1 cells. The uptake of FA-SCNPs in the presence of free folic acid (125 ng/mL) was also tested. (j, k) Time-dependent effects of FA-SCNPs on [Na^+^]_i_ (j) and chloride ([Cl^–^]_i_) (k), measured by SBFI-AM and MQAE, respectively, in both MOC1 and SCC1 cells. FA-SCNPs were incubated with the cells at 25 μg/mL. Saline at the same NaCl concentration or PBS were tested as controls. Fluorescence signals were normalized to that of untreated cells. Data are expressed as mean ± SD. Statistical difference was evaluated using a one-way ANOVA test. ****, *p* < 0.0001.

We then investigated the uptake of FA-SCNPs by MOC1 cells, a mouse oral squamous cell carcinoma cell line known to express the folate receptor.^17^ Rhodamine-labeled FA-SCNPs were internalized by the cancer cells, and their colocalization with Lysotracker indicated that the nanoparticles were primarily taken up via endocytosis (Figure 1g). This was further supported by the observation that sodium azide, a general endocytosis inhibitor, significantly reduced FA-SCNP uptake (Figure 1h). Moreover, coincubation with dynasore and chlorpromazine, inhibitors of dynamin- and clathrin-mediated endocytosis, respectively, also reduced uptake significantly (Figure 1h). In contrast, nystatin, a caveolar endocytosis inhibitor, had no significant effect. For comparison, SCNPs coated with DSPE-PEG(2000) Amine, but lacking DSPE-PEG(2000) Folate (PEG-SCNPs), showed relatively low uptake (Figure 1i and Figure S1). Meanwhile, the uptake of FA-SCNPs was effectively blocked by free folic acid (Figure 1i). These results indicate that FA-SCNPs are internalized via folate receptor-mediated endocytosis through the clathrin-dependent pathway.

We next investigated the effect of FA-SCNPs on intracellular sodium ([Na^+^]_i_) and chloride ([Cl^–^]_i_) levels using the fluorogenic probes SBFI-AM and MQAE, respectively. SBFI-AM fluorescence intensity correlates positively with intracellular sodium levels^18^ while MQAE fluorescence intensity correlates inversely with chloride levels.^19^ After incubating MOC1 cells with FA-SCNPs, SBFI-AM fluorescence increased within the first 2 h (Figure 1j), which is attributed to sodium release from degraded nanoparticles. This is supported by the observation that MQAE fluorescence significantly decreased during this period (Figure 1k), suggesting an increase in [Cl^–^]_i_. The increasing trend of SBFI-AM fluorescence was disrupted after 4 h, likely due to necrotic cell death caused by increased osmotic pressure, as previously reported.^20^ Similar trends in [Na^+^]_i_ and [Cl^–^]_i_ were observed in SCC1 cells, a human squamous cell carcinoma cell line (Figure 1j,k). For comparison, PBS and saline were also tested in both cell lines and showed minimal effects on intracellular sodium or chloride levels (Figure 1j,k).

We also assessed the impact of FA-SCNPs on intracellular calcium ([Ca^2+^]_i_), another critical cellular ion. Interestingly, both MOC1 and SCC1 cells exhibited a significant, time-dependent increase in [Ca^2+^]_i_ following incubation with FA-SCNPs (Figure 2a). Since FA-SCNPs do not contain calcium, this rise in [Ca^2+^]_i_ is likely an indirect consequence of altered intracellular sodium or chloride levels. To understand this, we repeated the incubation in calcium-free medium. Under this condition, the increase in [Ca^2+^]_i_ was minimal (Figure 2b), suggesting that the primary source of elevated [Ca^2+^]_i_ was extracellular calcium influx, rather than release from internal stores. Supporting this, treatment with BAPTA and EGTA, both of which are membrane-impermeable calcium chelators, effectively suppressed FA-SCNP-induced [Ca^2+^]_i_ elevation in regular medium (Figure 2b), further confirming that the observed calcium increase primarily originated from the extracellular space.

**Figure 2:**
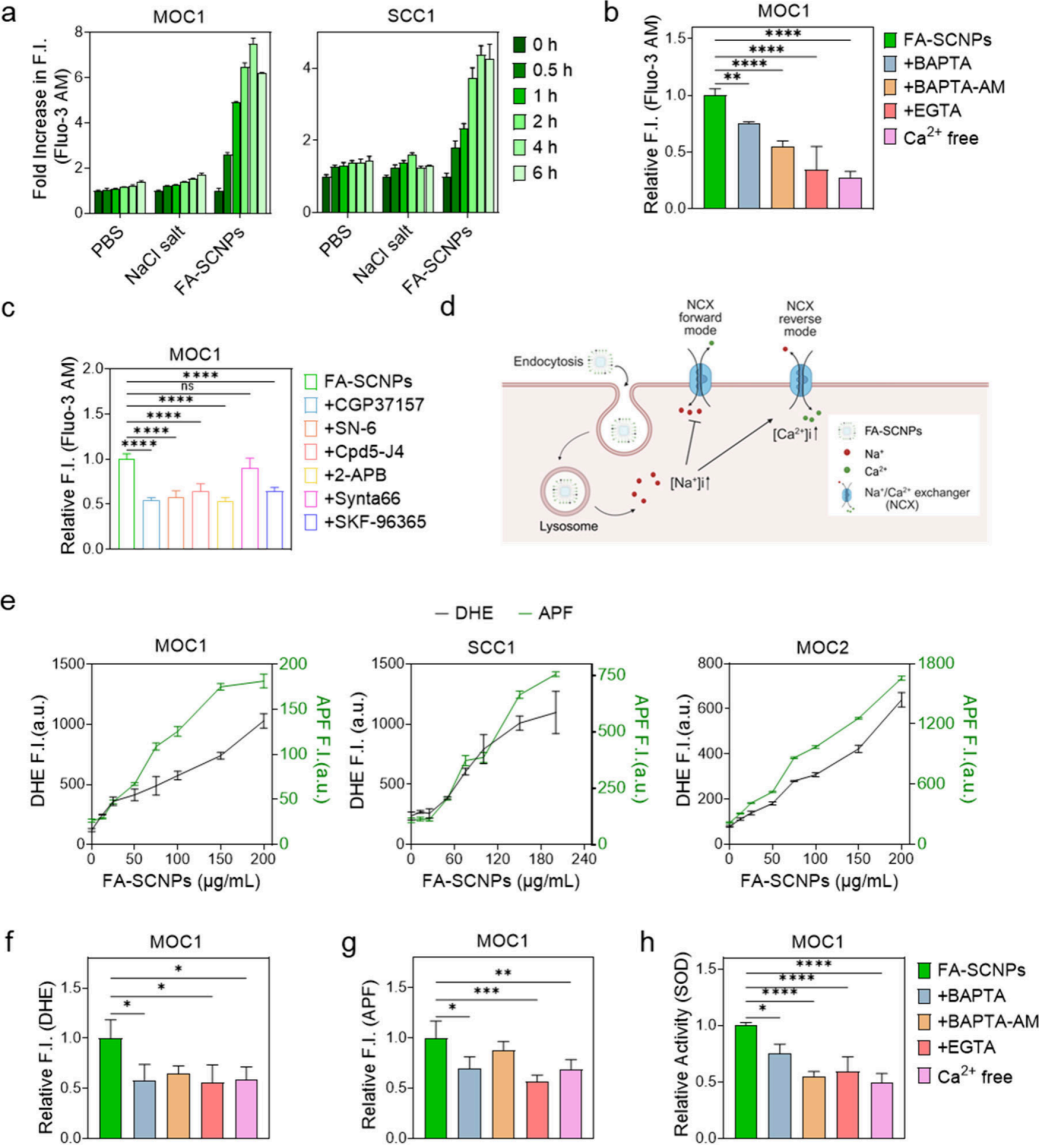
Influence of FA-SCNPs on intracellular calcium and ROS levels. FA-SCNPs were incubated with cells at 25 μg/mL. NaCl salt at the same concentration or PBS was tested for comparison. (a) Changes in [Ca^2+^]_i_ in MOC1 and SCC1 cells, measured using Fluo-3 AM at different time points. Fluorescence signals were normalized to those of untreated cells. (b) Relative [Ca^2+^]_i_ levels in MOC1 cells incubated with FA-SCNPs in the presence of calcium chelators (BAPTA, BAPTA-AM, and EGTA) or in calcium-free medium, evaluated using Fluo-3 AM. Fluorescence signals were normalized to cells treated with FA-SCNPs alone. (c) Relative [Ca^2+^]_i_ levels in MOC1 cells incubated with FA-SCNPs as well as calcium channel inhibitors (CGP37157, SN-6, Cpd5-J4, 2-APB, Synta66, SKF96365), measured using Fluo-3 AM. Fluorescence signals were normalized to cells treated with FA-SCNPs alone. (d) Scheme showing the mechanism underlying the calcium influx. FA-SCNPs enter cells through endocytosis and degrade inside cells to release large amounts of sodium. This leads to an increase in [Na^+^]_i_, forcing NCX to operate in a reverse mode, resulting in calcium influx. (e) Cellular levels of superoxide (black), assessed with DHE, and hydroxyl radical (green), assessed with APF, in MOC1, SCC1, and MOC2 cells treated with FA-SCNPs at various concentrations. (f, g) Relative levels of superoxide (f) and hydroxyl radicals (g) in MOC1 cells treated with FA-SCNPs in the presence of calcium chelators (BAPTA, BAPTA-AM, and EGTA) or in calcium-free medium. (h) SOD activity in MOC1 cells treated with FA-SCNPs, and then coincubated with calcium chelators (BAPTA, BAPTA-AM, and EGTA), or in calcium-free medium. Data are represented as mean ± SD. Statistical difference was evaluated using a one-way ANOVA test. n.s., not significant; *, *p* < 0.05; **, *p* < 0.01; ***, *p* < 0.001; ****, *p* < 0.0001.

Calcium influx in cells is tightly regulated, primarily through plasma membrane ion channels. To determine which specific channels mediate the FA-SCNP-induced calcium influx, we incubated cells with FA-SCNPs in the presence of various calcium-channel inhibitors, including CGP37157, SN-6, Cpd5-J4, 2-APB, Synta66, and SKF-96365 (Figure 2c). Synta66, an inhibitor of Orai1, specifically blocks CRAC channels,^21^ whereas SKF-96365 and 2-APB^22^ inhibit TRP cation channels. Our results revealed that SKF-96365 and 2-APB, but not Synta66, effectively suppressed the FA-SCNP-induced elevation of [Ca^2+^]_i_ (Figure 2c). These findings suggest that the observed calcium influx is at least partially mediated by TRP channels, a family of nonselective cation channels. It is known that some TRP channels, such as TRPV4, are sensitive to changes in osmolarity,^23^ supporting the possibility that increased intracellular sodium and chloride levels following FA-SCNP treatment activate TRP channels, leading to calcium influx.

Additionally, we observed that SN-6, a selective inhibitor of the sodium/calcium exchanger (NCX),^24^ also significantly reduced the FA-SCNP-induced increase in [Ca^2+^]_i_ (Figure 2c), indicating that NCX plays a substantial role in this process. Under normal physiological conditions, NCX typically exports calcium from cells in exchange for extracellular sodium.^25^ But it was found that at high intracellular sodium levels, NCX can function in a reverse mode, i.e., importing calcium into the cell in exchange for sodium.^26^ Thus, our results suggest that treatment with FA-SCNPs by elevating intracellular [Na^+^]_i_, forcing NCX to operate in reverse mode and consequently driving calcium influx (Figure 2d).

We then investigated the effect of FA-SCNPs on cellular oxidative stress. We observed a concentration-dependent increase in both superoxide and hydroxyl radical levels (Figure 2e). In contrast, the increase in radical levels was significantly reduced when the study was conducted in calcium-free medium (Figure 2f–h). Similarly, the use of calcium chelators such as BAPTA, EGTA, and BAPTA-AM (which sequester intracellular calcium), effectively suppressed the rise in ROS increase inside cells (Figure 2f–h). These findings suggest that FA-SCNPs can elevate oxidative stress in cells, and the impact is partially mediated by calcium influx. This observation aligns with previous reports that an uncontrolled and sustained [Ca^2+^]_i_ increase may cause calcium overload in mitochondria, leading to disrupted cell metabolism and consequently an increase of ROS level.^27–29^

Next, we investigated the interaction between FA-SCNPs and radiation. Radiation alone significantly increased superoxide and hydroxyl radical levels in both MOC1 and SCC1 cells (Figure 3a,b). Radiation also elevated [Ca^2+^]_i_ (Figure S2), which is consistent with previous reports;^30^ this effect is attributed to the activation of surface ion channels, such as TRPM2 and TRPV4, which are known to respond to oxidative stress.^31,32^ Combining radiation with FA-SCNP treatment further amplified superoxide and hydroxyl radical production (Figure 3a,b), suggesting that FA-SCNPs potentiate radiation-induced oxidative stress. This enhanced ROS generation was significantly reduced by cotreatment with EGTA (Figure S3a,b), indicating that calcium influx, driven by both FA-SCNPs and radiation, plays an important role in mediating the elevated oxidative stress.

**Figure 3:**
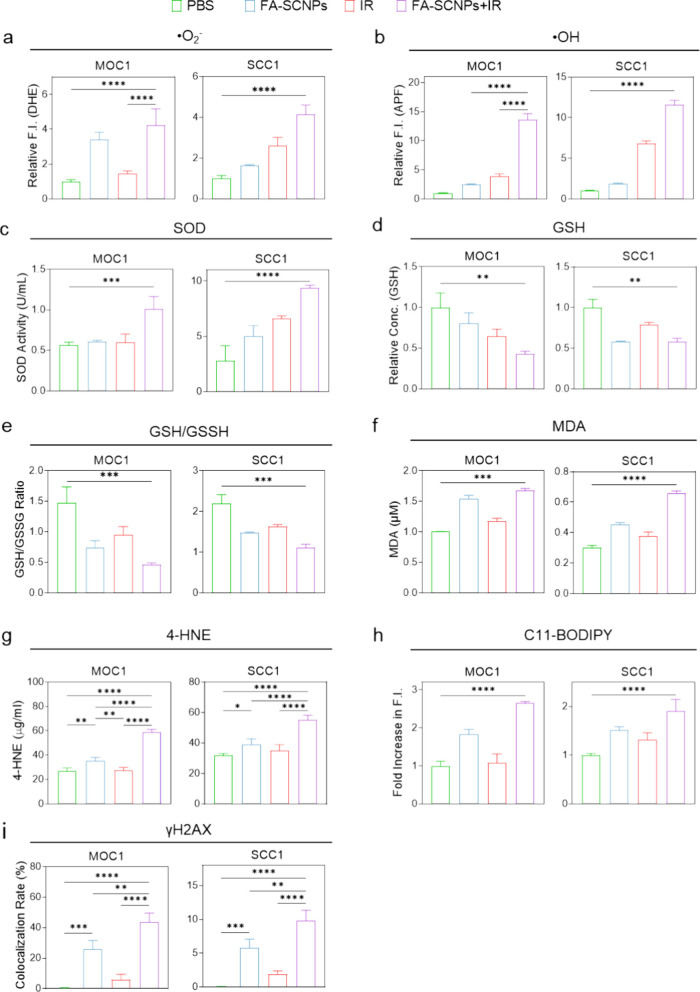
*In vitro* evaluation of the radiosensitizing effects of FA-SCNPs. FA-SCNPs (25 μg/mL) were incubated with cancer cells for 4 h prior to irradiation (IR, 5 Gy). For comparison, FA-SCNPs alone, IR alone, or PBS alone were also tested. Experiments were conducted with both MOC1 and SCC1 cells. (a, b) Cellular levels of superoxide (a), measured using DHE, and hydroxyl radicals (b), measured using APF, in MOC1 and SCC1 cells. (c) SOD activity in MOC1 and SCC1 cells. (d, e) Relative levels of GSH and GSH/GSSG ratios in MOC1 and SCC1 cells. (f) MDA levels, measured by TBARS assay. (g) 4-HNE levels in cancer cells, determined by 4-HNE assay. (h) Lipid peroxidation, assessed by C11-BODIPY assay. (i) DNA damage, measured by γH2AX assay. Data are represented as mean ± SD. Statistical difference was evaluated using a one-way ANOVA test. *, *p* < 0.05; **, *p* < 0.01; ***, *p* < 0.001; ****, *p* < 0.0001.

To further characterize the oxidative stress response, we measured superoxide dismutase (SOD) activity and glutathione (GSH) levels in SCC1 and MOC1 cells. SOD catalyzes the dismutation of superoxide radicals into hydrogen peroxide, while GSH is a crucial antioxidant that maintains cellular redox balance. Upon combined treatment with FA-SCNPs and radiation, we observed a significant reduction in the ratio of GSH to oxidized glutathione (GSSG) (Figure 3d,e), indicating GSH depletion due to elevated oxidative stress. Concurrently, a marked increase in SOD activity was detected (Figure 3c), likely representing a cellular protective response to the increased ROS levels.

Elevated oxidative stress can damage critical cellular components, including DNA and lipids. To assess this damage, we performed γ-H2AX staining for DNA double-strand breaks and lipid peroxidation assays, including TBARS, 4-hydroxynonenal (4-HNE), and C11-BODIPY assays. FA-SCNP treatment, alone or combined with radiation, significantly increased lipid peroxidation, as evidenced by elevated levels of MDA (Figure 3f), 4-HNE (Figure 3g), and C11-BODIPY fluorescence (Figure 3h). Similarly, FA-SCNPs alone induced notable DNA double-strand breaks, which were further augmented by combined irradiation (Figure 3i).

**Figure 4:**
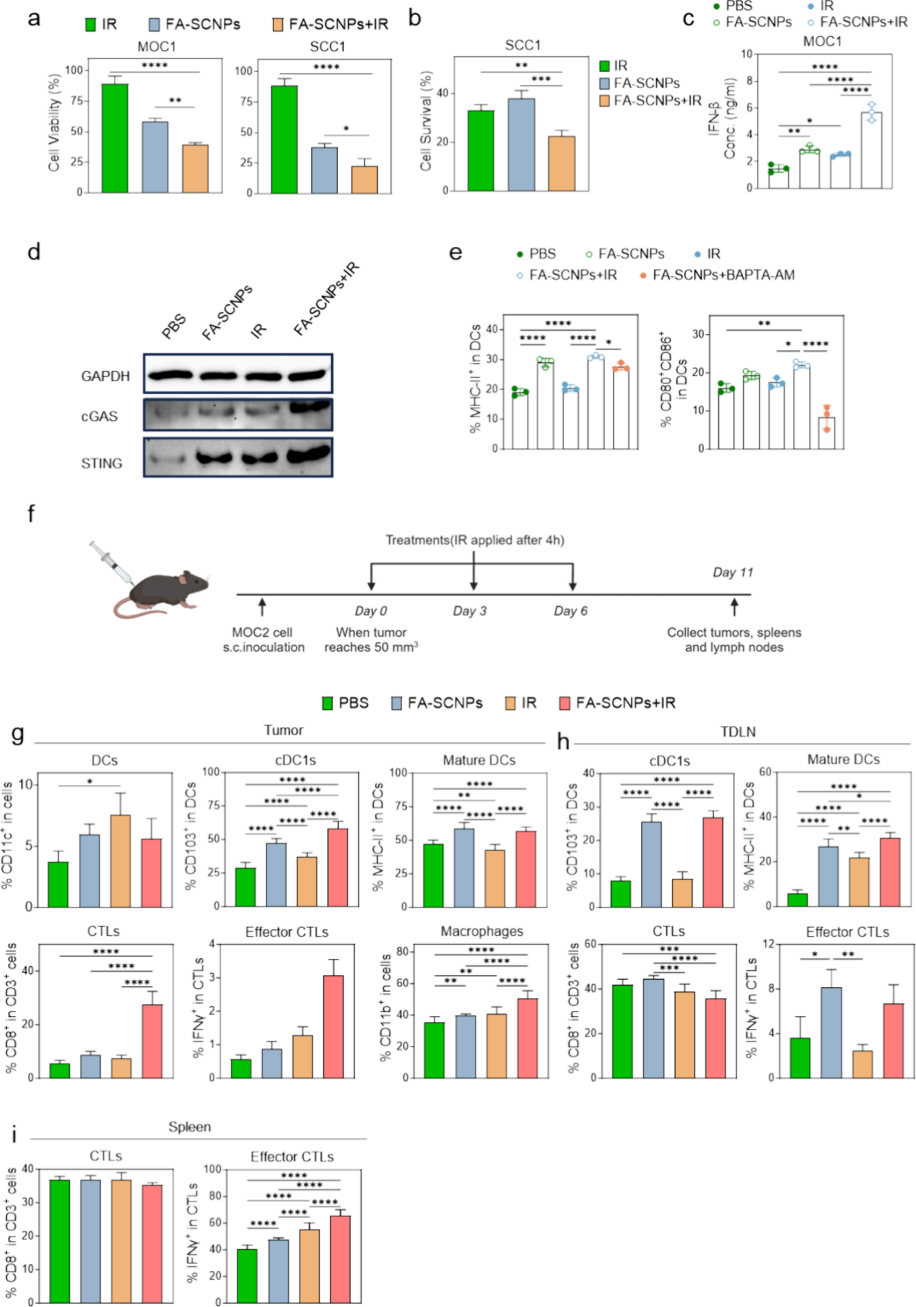
FA-SCNPs combined with radiation induces immunogenic cell death. FA-SCNPs (25 μg/mL) were incubated with cancer cells, with or without irradiation (IR, 5 Gy) after 4 h. (a) Cytotoxicity, measured by MTT assay at 24 h in MOC1 and SCC1 cells. (b) Clonogenic survival, measured by clonogenic assay in SCC1 cells at 2 weeks. (c) IFN-β secretion, measured by ELISA in MOC1 cells at 24 h. (d) STING and cGAS activation, assessed by Western blotting in MOC1 cells. (e) Influence on DC maturation. MOC1 cells were treated with FA-SCNPs (25 μg/mL) plus IR (5 Gy), in the presence or absence of BAPTA-AM, and then cocultured with BMDCs. FA-SCNPs alone, IR alone, or PBS alone were tested for comparison. Flow cytometry was performed to measure the percentages of MHC-II^+^ and CD80^+^CD86^+^ cells among DCs. (f-i) Influence on the tumor microenvironment, evaluated in MOC2-tumor-bearing C57BL/6 mice. FA-SCNPs (3.25 mg/mL, 30 μL) was administered intratumorally, and tumor IR (5 Gy) was applied at 4 h. For comparison, PBS, FA-SCNPs alone, and IR alone were also tested. Animals were euthanized on Day 5. Tumor, TDLN, and spleen were collected for flow cytometry analysis. (f) Schematic illustration of the experimental plan. (g-i) Frequencies of DCs (CD11c^+^), cDC1s (CD103^+^CD11c^+^), mature DCs (MHC-II^+^CD11c^+^), CTLs (CD3^+^CD8^+^), effector CTLs (IFN-γ^+^CD3^+^CD8^+^), and myeloid cells (CD11b^+^) in (g) tumor, (h) TDLN, and (i) spleen. Data are presented as mean ± SD. Statistical difference was evaluated using a one-way ANOVA test. *, *p* < 0.05; **, *p* < 0.01; ***, *p* < 0.001; ****, *p* < 0.0001.

Next, we evaluated whether FA-SCNPs could sensitize cancer cells to radiation-induced cell death. MTT assay revealed moderate decreases in cell viability at 24 h following treatment with radiation alone (5 Gy) (Figure 4a). Combining FA-SCNPs with radiation significantly enhanced cancer cell killing (Figure 4a). Synergy factor (SF) values were below 1 for both SCC1 (0.67) and MOC1 (0.76) cells, indicating a synergistic interaction between FA-SCNPs and radiation. Consistently, clonogenic assays also demonstrated good synergy between radiation and FA-SCNPs at reducing colony formation (Figure 4b). Together, these findings confirm that FA-SCNPs synergize with radiation to enhance cancer cell killing and inhibit proliferation. Notably, cotreatment with the NCX inhibitors diltiazem and CGP37157 moderately but significantly attenuated cell death caused by combined FA-SCNP and radiation treatment (Figure S4a,b), supporting our earlier observation that calcium influx plays a role in mediating the cytotoxic effects. Meanwhile, aged FA-SCNPs exhibited no radiosensitizing activity (Figure S5), as degraded nanoparticles lose the ability of modulating intracellular osmotic and oxidative pressure.

In addition to directly killing tumor cells, radiation has been reported to enhance antitumor immune responses. One key mechanism behind the stimulatory effect involves the radiation-induced release of double-stranded DNA (dsDNA) from the nucleus into the cytosol, which subsequently activates the cGAS-STING pathway.^33^ This activation triggers increased secretion of type I interferons, which stimulate antigen-presenting cells, particularly DCs, thereby promoting cross-presentation and enhancing cellular immunity.^34^ Recent studies suggest that calcium signaling plays a critical role in promoting STING phosphorylation and activation.^35,36^ Based on this, we hypothesized that FA-SCNPs could enhance the immunostimulatory effects of radiation.

Consistent with this hypothesis, we found that the combination of FA-SCNPs and radiation significantly increased IFN-β secretion compared to radiation alone (Figure 4c). Western blot analysis showed elevated expression of cGAS and STING, supporting the notion that increased IFN-β secretion is driven by activation of the cGAS-STING pathway (Figure 4d). To further evaluate the immune-stimulatory effects, we treated MOC1 cells with PBS, FA-SCNPs alone, radiation alone, or their combination, and then cocultured the treated cancer cells with bone marrow-derived dendritic cells (BMDCs). Cancer cells treated with the combination induced greater DC maturation compared to radiation alone, as evidenced by increased populations of MHC-II^+^ and CD80^+^CD86^+^ DCs (Figure 4e). Notably, the addition of BAPTA-AM significantly reduced the number of mature DCs, indicating that this activation was at least partially mediated by FA-SCNP-induced calcium influx.

We then evaluated the immunogenicity of this approach in vivo (Figure 4f). This was evaluated in MOC2 tumor bearing mice, which are more aggressive and less responsive to radiotherapy or immunotherapy compared to MOC1 tumors.^37^ The combination of FA-SCNPs and radiation significantly increased the number of mature DCs within the tumors (Figure 4g). In particular, the frequency of conventional type 1 DCs (cDC 1s, CD103^+^ DCs), a subset critical for antigen cross-presentation,^38^ was markedly increased (Figure 4g). This was accompanied by elevated levels of mature DCs and cDC 1s in tumor-draining lymph nodes (TDLNs) (Figure 4h), suggesting enhanced DC migration. In parallel, we observed increased infiltration of T cells, particularly cytotoxic T lymphocytes (CTLs) and effector (IFN-γ^+^) CTLs, within the tumors (Figure 4h). Enhanced effector CTL populations were also detected in the spleen (Figure 4i). Collectively, these findings suggest that FA-SCNPs amplify radiation-induced immune responses, leading to improved priming and expansion of tumor-reactive T cells.

Finally, we evaluated the therapeutic benefits of combining FA-SCNPs with radiotherapy. This was first evaluated in C57BL/6 mice bearing flank MOC1 tumors. When tumors reached approximately 50 mm^3^, mice received intratumoral injections of FA-SCNPs (3.25 mg/mL, 30 μL). Four hours later, the tumors were irradiated (Figure 5a). A total of three treatments were administered at three-day intervals. Control groups received PBS, radiation alone, or FA-SCNPs alone. Compared to PBS, both FA-SCNPs and radiation moderately suppressed tumor growth (Figure 5b,e). In contrast, the combination treatment resulted in significant tumor suppression, reducing tumor size by 81.5% by Day 52 (Figure 5b). By Day 80, 60% of the mice in the combination group remained alive and tumor-free (Figure 5c). Importantly, surviving animals rejected tumor rechallenge with live MOC1 cells, indicating the development of tumor-specific immune memory. However, depletion of CTLs using an anti-CD8 antibody nearly abolished the therapeutic benefit (Figure 5b,c,e), indicating that cellular immunity plays a critical role in tumor control and long-term survival beyond the direct tumoricidal effects of the treatment.

**Figure 5:**
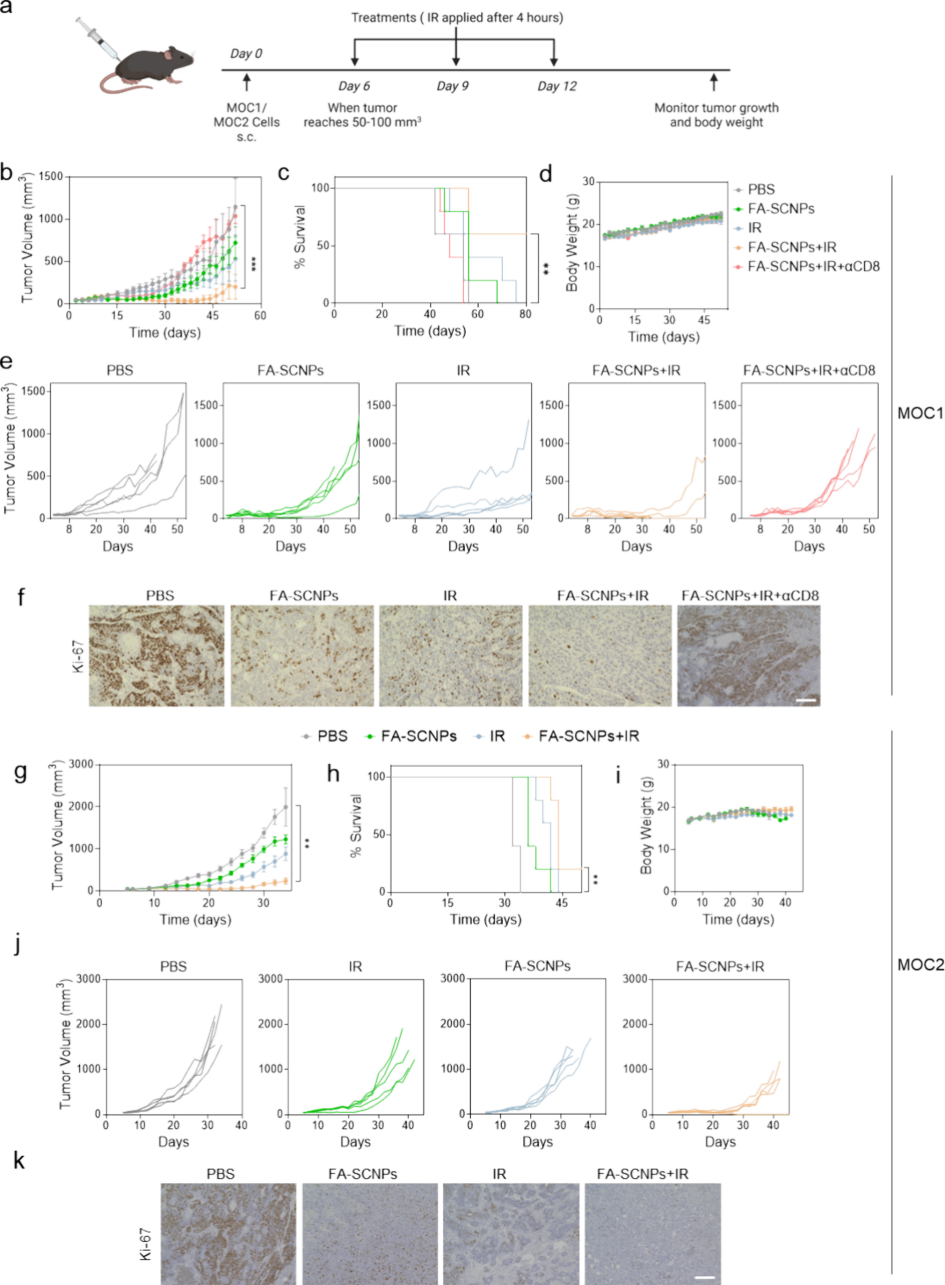
*In vivo* evaluation of the radiosensitizing effects of FA-SCNPs. (a) Schematic illustration of the experimental plan. FA-SCNPs (3.25 mg/mL, 30 μL) was administered intratumorally, and tumor irradiation (IR, 5 Gy) was applied at 4 h. A total of three treatments were administered at 3-day intervals. The experiments were performed in both MOC1 and MOC2 tumor-bearing C57BL/6 mice. In MOC1 tumor models, the combination therapy was also tested in animals depleted of CTLs using anti-CD8 antibodies. PBS, FA-SCNPs, and IR alone were tested in both tumor models for comparison. (b-f) Therapeutic evaluation results in MOC1-tumor-bearing mice. (b) Average tumor growth curves. Statistical difference was evaluated using a one-way ANOVA test. **, *p* < 0.01. (c) Animal survival curves. Statistical difference between the PBS and FA-SCNP+IR groups were calculated by the log-rank Mantel-Cox test. **, *p* < 0.01. (d) Average animal body weight curves. (e) Individual tumor growth curves. (f) Ki67 staining of tumor tissues. (g-k) Results from tests in MOC2-tumor-bearing mice. (g) Average tumor growth curves. Statistical difference was evaluated using a one-way ANOVA test. **, *p* < 0.01. (h) Average animal survival curves. Statistical difference was computed between the PBS and FA-SCNP+IR groups using the log-rank Mantel-Cox test. **, *p* < 0.01. (i) Average animal body weight curves. (j) Individual tumor growth curves. (k) Ki67 staining of tumor tissues. Data are represented as mean ± SEM.

When evaluated in MOC2 tumor bearing mice, neither FA-SCNPs nor radiation alone significantly suppressed tumor growth (Figure 5g,j). As a comparison, their combination effectively arrested tumor growth during the first 3 weeks of treatment. While some tumors eventually relapsed, 20% of the mice achieved complete tumor remission (Figure 5g,h,j). Importantly, no acute toxicity or weight loss was observed in either model throughout the study (Figure 5d,i). Histological analysis revealed reduced Ki67 staining in tumors treated with the combination therapy from both models (Figure 5f,k). Additionally, no pathological abnormalities were observed in major organs (Figure S6), indicating good treatment tolerability.

Taken together, these results demonstrate that FA-SCNPs safely and effectively enhance the therapeutic efficacy of radiation in both responsive and resistant tumor models.

In this study, we evaluated FA-SCNPs as a radiosensitizer using head and neck cancer models. Our findings demonstrate that FA-SCNPs significantly enhance radiation-induced cancer cell death and stimulate antitumor immunity, leading to improved tumor control and prolonged animal survival. Unlike conventional nanoparticle-based radiosensitizers that rely on high-Z elements, FA-SCNPs function by disrupting cellular osmotic and oxidative balances, thus offering an alternative mechanism of radiosensitization.

The current FA-SCNPs formulation is designed for local injection to enhance radiotherapy, and our studies indicate that FA-SCNPs are largely retained within tumors after administration (Figure S7). This intratumoral approach is analogous to NBTXR3, which remains the only inorganic nanoparticle radiosensitizer currently in clinical use. Because radiotherapy is often delivered locally, intratumoral administration of radiosensitizers is clinically relevant for multiple malignancies, including head and neck cancers, which are the focus of the present work. In the future, it is possible to develop formulations suitable for systemic administration; however, this will require further optimization to balance nanoparticle stability, delivery efficiency, and maximal modulation of cellular osmotic and oxidative balance in cancer cells.

While radiation is known to stimulate antitumor immune responses, it is often insufficient to elicit a robust immune activation on its own. Recent studies have identified the cGAS-STING pathway and the subsequent release of type I interferons as key mediators of radiation-induced immune stimulation.^39^ However, this effect is limited by the radiation-induced activation of Trex1, a DNA exonuclease that degrades cytosolic DNA, thereby dampening cGAS-STING activation at higher radiation doses.^40^ Our data suggest the potential of combining radiation with FA-SCNPs, which enhances the release of tumor-associated antigens through direct cell killing and amplifies the cGAS-STING signaling by modulating cellular calcium levels. Together, these effects strengthen cellular immune responses and improve therapeutic outcomes.

Future studies will focus on further elucidating the mechanisms underlying this synergy and optimizing the dosing and scheduling of FA-SCNPs and radiation to maximize their combined effects on local tumor control and systemic immunity. The current study employed a fraction dose of 5 Gy, which is common in small-animal models.^41–44^ It will be worthwhile to investigate alternative hypofractionation schemes to determine how different dosing combinations influence radio-immunotherapy outcomes. Furthermore, exploring combination regimens that integrate FA-SCNPs, radiotherapy, and immune checkpoint blockade could further enhance antitumor immune responses and promote durable, long-term survival in cancer patients. Our approach may also be applicable to other electrolyte-based nanoparticles which may similarly disrupt ionic homeostasis or redox balance within tumor cells. In this context, the present findings suggest a broader design framework for leveraging nanomaterials in radiation therapy.

## Supplementary Material



## Notes

Materials and methods; cellular uptake of nanoparticles; effect of irradiation on intracellular calcium levels; *in vitro* evaluation of the radiosensitizing effects of FA-SCNPs; effect of FA-SCNPs with NCX inhibitors on cell viability and LDH release; radiosensitizing effects of fresh versus aged FA-SCNPs; H&E staining images; comparison of the radiosensitizing effects of fresh versus aged FA-SCNPs; distribution of SCNPs; additional reference (PDF)
