# Supplementary material for: Sodium Chloride Nanoparticles Potentiate Radiation Therapy by Disrupting Osmolarity Balance and Enhancing Antitumor Immunity
Source: Nano Lett. 2025 Sep 15;25(38):14032–42. doi: 10.1021/acs.nanolett.5c03022 (PMC12464967; doi:10.1021/acs.nanolett.5c03022)
Supplement: Supplementary file 1 [file nl5c03022_si_001.pdf]

Supporting Information for

Sodium Chloride Nanoparticles Potentiate Radiation Therapy by Disrupting Osmolarity Balance and Enhancing Anti-Tumor Immunity

Shuyue Zhan[1‡], Jianwen Li[1‡], Xinning Lai[1‡], Yaochao Zheng[2], Zhizi Feng[1], Sahil Bakul Patel,[1] Wei Yang[1], Yong Teng[3], Yao Yao[2], Jin Xie[1,4,5]*

[1] Department of Chemistry, University of Georgia, Athens, GA 30602, USA.

[2] Regenerative Bioscience Center, Department of Animal and Dairy Science, University of Georgia, Athens, GA 30602, USA

[3] Department of Hematology and Medical Oncology & Winship Cancer Institute, Emory University School of Medicine, Atlanta, GA 30322, USA

[4] Department of Pharmaceutical & Biomedical Sciences, University of Georgia, Athens, GA 30602, USA

[5] School of Chemical, Materials and Biomedical Engineering, College of Engineering, The University of Georgia, Athens, Georgia, USA

‡ These authors contributed equally to this work.

* Corresponding author

Emails: jinxie@uga.edu

Supporting Information includes:

1. Materials and methods

2. Supplementary Figures

3. Reference

Materials and methods

Nanoparticle synthesis

Synthesis of sodium chloride nanoparticles (SCNPs). Typically, at room temperature, 213 mg of sodium acetate (anhydrous, 99%, Sigma-Aldrich) was first completely dissolved in 65 mL of ethanol (PURE 200 PROOF, KOPTEC). 20 mL of oleylamine (70%, Sigma-Aldrich), which serves as a surfactant, and 10 mL of hexane (99.9%, Fisher), were then added to the mixture. Using magnetic stirring, 8 mL of hexane containing 160 μL of acetyl chloride (99.0%, Sigma-Aldrich) was added dropwise over 1.5 minutes using a 1 mL pipette. After the last drop, the SCNPs were immediately collected by centrifugation at 10,000 rpm for 3 minutes and washed with 20 mL of isopropanol to remove unreacted precursors. Finally, the nanoparticles were redispersed in isopropanol and dried by rotary evaporation (Buchi R II Rotavapor). The SCNPs were sealed with parafilm and carefully stored in a desiccator to avoid humidity.

Synthesis of folate-coated sodium chloride nanoparticles (FA-SCNPs). Ten milligrams of as-synthesized nanoparticles were dispersed in 1.5 mL of isopropanol and sonicated for two minutes. Then, 0.45 mg of DSPE-PEG(2000) amine (1,2-distearoyl-sn-glycero-3-phosphoethanolamine-N-[amino(polyethylene glycol)-2000] (ammonium salt), Avanti) and 0.05 mg of DSPE-PEG(2000) folate (1,2-distearoyl-sn-glycero-3-phosphoethanolamine-N-[folate(polyethylene glycol)-2000] (ammonium salt), Avanti) were added, and the mixture was sonicated for an additional 30 seconds. The final product was dried by rotary evaporation. For Rhodamine B-labeled FA-SCNPs (RB-FA-SCNPs), 16:0 Liss Rhod PE (1,2-dipalmitoyl-sn-glycero-3-phosphoethanolamine-N-(lissamine-rhodamine B sulfonyl) (ammonium salt), Avanti) was added during sonication, while all other steps remained the same. For Cy5.5 labeling, a Cy5.5-DSPE-PEG(2000) conjugate was first synthesized by NHS-amine coupling of Cy5.5 NHS ester (Lumiprobe, 17020) to DSPE-PEG(2000) amine, as previously described.[1] The conjugate was then incorporated during FA-SCNP assembly. All other steps remained the same.

Nanoparticle characterization

Nanoparticle images were captured by transmission electron microscopy (TEM) using a STEM-Hitachi SU9000EA instrument operated at 30 kV and 2 μA. Elemental mapping images via energy dispersive X-ray spectroscopy (EDS) were acquired with a TEM (STEM-Hitachi SU9000EA)-compatible Oxford EDS system. The hydrodynamic sizes and zeta potentials of the nanoparticles were measured using a Malvern Zetasizer Nano ZS system.

Cell culture

The human head and neck squamous cell carcinoma cell line SCC1 were obtained from the University of Michigan. The mouse oral cavity squamous cell carcinoma cell lines MOC1 and MOC2 were purchased from Kerafast (Boston, MA). Cells were cultured in DMEM (ATCC, 30-2002) medium supplemented with 10% fetal bovine serum (FBS, Atlanta Biologicals, S11150) and 1% penicillin/streptomycin (Gibco, 1140-122). Bone marrow derived dendritic cells (BMDCs) were established from the bone marrow of C57BL/6 mice and cultured in RPMI-1640 (Corning, 10-040-CV) containing GM-CSF (PeproTech®, 315-03) according to a published protocol.[2] All cells were maintained in a humidified atmosphere at 37 °C with 5% CO_2 (HERAcell 150, Thermo Scientific).

***In vitro* cellular uptake**

In vitro cellular uptake was investigated with fluorescence microscopy (Keyence, BZ-X810) and flow cytometry. For fluorescence microscopy, MOC1 cells were seeded in 6-well plates (Corning, 3516) at a density of 5×10^5 cells per well and incubated overnight. The RB-FA-SCNPs (25 μg/mL) were then added and incubated for 4 hours. The medium was removed after incubation. The cells were washed thrice with PBS and fixed with 4% paraformaldehyde for 15 minutes, and the cell nuclei were stained with 1 μg/mL DAPI (Thermo Fisher, D1306) for 5 minutes. To visualize the lysosomes, cells were incubated with RB-FA-SCNPs (25 μg/mL) for 4 hours, followed by staining with 50 nM LysoTracker (Thermo Fisher, L7526) for 30 minutes. The cells were then washed thrice with PBS and observed using fluorescence microscopy.

For the flow cytometry measurements, SCC1 or MOC1 cells, at a density of 5×10^5 cells per well, were seeded in 6-well plates. To investigate the cellular uptake pathway, chemical endocytosis inhibitors, including sodium azide (NaN_3) (50 mM, Sigma, S2002), Dynasore (80 µM, Abcam, ab120192), Nystatin (25 µM, Sigma, N6261), and Chlorpromazine (100 µM, Sigma, C8138), were added prior to nanoparticle treatment. After 1 hour of pre-treatment, RB-FA-SCNPs were added for further incubation for 4 hours. Cells were harvested by cell lifter, washed with PBS, stained with DAPI and fixed with IC fixation buffer (eBioscience, 00-8222-49)/staining buffer (eBioscience, 00-4222-57). Trypan blue solution (0.4%, GibcoTM) was added to quench fluorescence from cell surface membrane-bound nanoparticles at a final concentration of 20 µg/mL. Intercellular RB-FA-SCNPs signals were detected by flow cytometry (NovoCyte Quanteon Flow Cytometer Systems 4 Lasers) and analyzed for mean fluorescence intensity (MFI).

Cellular ion concentrations

SCC1 or MOC1 cells were seeded into a 96-well black plate (Costar, 3610) at a density of 6,000 cells per well and incubated overnight. Cells were loaded with 10 µM SBFI-AM (sodium binding benzofuran isophthalate acetoxymethyl ester, Setareh Biotech, 6212), 10 mM MQAE (1-(ethoxycarbonylmethyl)-6-methoxyquinolinium bromide, Setareh Biotech, 6270), or 10 µM Fluo-3 AM (Invitrogen™, F1241) in serum-free medium (as serum may contain esterase activity) containing 0.02% Pluronic® F-127 (Sigma, P2443) for 30 minutes before the addition of FA-SCNPs. For comparison, inhibitors including BAPTA (10 µM, Invitrogen™, B1204), BAPTA-AM (10 µM, Sigma, 196419), EGTA (50 µM, Sigma, 324626), Diltiazem (50 µM, Thermo Scientific, 329080050), CGP37157 (10 µM, Sigma, C8874), SN-6 (5 µM, Tocris Bioscience™, 2184/10), Cpd5-J4 (10 µM, Sigma, 5306470001), 2-APB (10 µM, Sigma, 100065), Synta66 (5 µM, Sigma, SML1949), and SKF-96365 (5 µM, Sigma, S7809) were pre-treated with cells for 1 hour prior to nanoparticle treatment. Fluorescence signals were recorded at different time points (0, 0.5, 1, 2, 4, 6 hours) using a microplate reader (Synergy Mx, BioTeK) (SBFI-AM: E_x/E_m= 339 nm/565 nm, MQAE: E_x/E_m = 350 nm/460 nm, Fluo-3 AM: E_x/E_m = 506 nm/526 nm). PBS and NaCl salt were used as controls.

Cell studies

The effect of FA-SCNPs on oxidative stress, DNA damage, lipid peroxidation and cell viability was assessed in SCC1 and MOC1 cells.

To investigate the ROS production, SCC1 or MOC1 cells were seeded in a black 96-well plate (Costar, 3610) at a density of 6,000 cells per well. After incubation overnight, the cells were treated with FA-SCNPs at different concentrations for 4 hours. To examine the combined effect of radiation and FA-SCNPs on ROS production, the cells were treated with FA-SCNPs (25 μg/mL) for 4 hours. After incubation, the medium was changed, and the cells were exposed to 5 Gy of radiation for another one-hour incubation. Superoxide ($O_2^{\cdot -}$) generation was detected after treatment using a dihydroethidium assay kit (DHE, Cayman chemical, 601290) according to the manufacturer protocol. Hydroxyl radicals (·OH) generation was detected using aminophenyl fluorescein (APF, Invitrogen, A36003) according to the manufacturer's instructions. For comparison, inhibitors such as BAPTA (10 μM, Invitrogen™, B1204), BAPTA-AM (10 μM, Sigma, 196419) and EGTA (50 μM, Sigma, 324626) were incubated with cells for 1 hour before nanoparticles treatment.

To investigate the antioxidant activities, the activity of superoxide dismutase (SOD) and the level of glutathione (GSH) and oxidized glutathione (GSSG) was assessed in SCC1 and MOC1 cells. Briefly, SCC1 or MOC1 cells were seeded in a 6-well plate (Costar, 3516) at a density of 0.5 M per well and incubated overnight. The cells were treated with FA-SCNPs (25 μg/mL) for 4 hours and then irradiated with 5 Gy radiation if applicable. After another one-hour incubation, cells were collected with a cell lifter and washed with PBS. Total SOD activity (cytosolic and mitochondrial) was measured using a SOD assay kit (Cayman chemical, 706002). GSH and GSSG levels were quantified using a GSH assay kit (Cayman chemical, 703002) according to the manufacturer's protocol.

DNA damage was accessed using an anti-γH2AX antibody (Biolegend, Alexa 488, 613405). SCC1 or MOC1 cells were seeded in a 4-well cell culture chamber slide (Falcon, 354114) at a density of 0.1 M per well and incubated overnight. Cells were treated as described above. Then cells were washed with PBS, stained with the anti-γH2AX antibody (1:500 dilution), and covered with DAPI mount medium. Images were acquired by an all-in-one fluorescence microscope

(KEYENCE BZ-X800). Quantification of DAPI and γH2AX colocalization was analyzed using ImageJ software.

To investigate the effect of FA-SCNPs on lipid peroxidation and cell viability, SCC1 or MOC1 were seeded in a 96-well plate and incubated overnight. Cells were treated as described above. Lipid peroxidation was assessed by measuring malondialdehyde (MDA) using a TBARS assay kit (Cayman chemical, 10009055) according to the manufacturer's protocol. Additionally, 4-HNE was detected using the 4-HNE assay kit (Abcam, 238538). BODIPY lipid was assessed using C11-BODIPY (Invitrogen, D3861) according to the manufacturer's protocol. In addition, cytotoxicity was assessed 24 hours post-treatment using the MTT assay (Sigma-Aldrich, M2128). The synergy factor (SF) between FA-SCNPs and irradiation was calculated as follows: $SF=AB/(A\times B)$, where A represents the SF ratio between the FA-SCNPs group and the PBS group, B represents the SF ratio between the irradiation group and the PBS group, and AB represents the SF ratio of combined treatment (irradiation plus FA-SCNPs) to the PBS group. SF values of <1, =1, or >1 indicate synergistic, additive, or antagonistic effects, respectively. LDH release was measured 24 hours post-treatment using the LDH Assay Kit-WST (Dojindo, CK12-20).

Clonogenic assays were performed in SCC1 cells. Briefly, SCC1 cells were seeded in a 6-well plate (Costar, 3516) at a density of 0.5 M per well and incubated overnight. Cells were treated with FA-SCNPs (25 μg/mL) for 4 hours, followed by 5 Gy irradiation. Cells were then cultured for an additional 20 hours. For comparison, a radiation-only group was included. Afterward, cells were collected, counted, and reseeded into a 6-well plate at a density of 100-500 cells per well, followed by a 14-day culture. The colonies were stained with crystal violet and counted.

To investigate BMDCs' maturation, BMDCs were seeded onto a 6-well plate at a density of 1×10^6 cells per well. BMDCs were treated with FA-SCNPs (25 μg/mL) for 4 hours and then irradiated with 5 Gy radiation if irradiation was applied. After incubation for another 20 hours, the BMDCs were harvested and were subsequently stained with MHCII (Biolegend, 107616), CD80 (eBioscience™, 46-0801-82), and CD86 (Biolegend, 105106) and analyzed by flow cytometry. For comparison, BAPTA (10 μM, Invitrogen™, B1204) was added to BMDCs 1 hour prior to FA-SCNPs treatment.

Western blotting

MOC1 cells were seeded onto a 6-well plate at a density of 0.5×10^6 cells per well. MOC1 were treated with FA-SCNPs (25 μg/mL) for 4 hours and then irradiated with 5 Gy radiation if irradiation was applied. After incubation for another 20 hours, cells were lysed using RIPA buffer (Thermo Scientific, 89901) supplemented with 100 × Halt^{TM} Proteinase and Phosphatase Inhibitor Cocktail (Thermo Scientific, 78445). Protein concentrations in the cell lysates were measured using a DC protein assay kit (BioRad). Equal amounts of protein lysates were loaded onto a 10% SDS-PAGE gel and transferred to PVDF membranes. To block nonspecific binding, membranes were incubated with 5% bovine serum albumin (BSA, Sigma) for 1 hour at room temperature. Membranes were then incubated with primary antibodies overnight at 4 °C with gentle shaking. After washing, membranes were incubated with secondary antibodies for 1 hour at room temperature. Membranes were incubated with Clarity Max Western ECL Substrate (BioRad, 1705062) and exposed to X-ray films (Biorad, ChemiDoc^{TM}) for signal detection. Equal protein loading was confirmed by probing for GAPDH. Antibodies used for detection were STING (Cell Signaling, 50494, 1:1000 dilution), cGAS (Cell Signaling, 31659, 1:1000 dilution), and GAPDH (Cell Signaling, 2118, 1:1000 dilution).

Cytokine release

MOC1 cells were seeded into 24-well plates at a density of 5×10^4 cells per well and incubated overnight. The cells were treated with FA-SCNPs (25 μg/mL) for 4 hours, followed by 5 Gy irradiation, and maintained for another 20 hours. For comparison, radiation-only treatment was tested. After incubation, the supernatants were collected and centrifuged at 1000 g for 10 minutes. Cellular release of IFN-β was then measured by using Mouse ELISA Kit (R&D, DY8234-05) following the manufacturer's protocol.

***In vivo* studies**

Animals. All experimental procedures were conducted in accordance with protocols approved by the Institutional Animal Care and Use Committee (IACUC) of the University of Georgia. C57BL/6 mice (female, 4 weeks old) were purchased from Envigo Laboratories and maintained under pathogen-free conditions. The animal models were established by subcutaneously injecting 1×10^6 MOC1 or MOC2 cells in 50 μL PBS into the right hind limb of each mouse after 2 weeks of settlement (6 weeks old).

Flow cytometry. C57BL/6 mice bearing MOC2 tumors were randomly divided into four groups (n = 5 for each group) and treated with (1) PBS (30 μL), (2) FA-SCNPs (3.25 mg, 30 μL) in PBS, (3) 5 Gy irradiation, and (4) FA-SCNPs (3.25 mg, 30 μL) + 5 Gy irradiation, respectively. The treatment began when the tumor size reached ~50 mm^3 (Day 0). FA-SCNPs was administered intratumorally, and tumor irradiation (5 Gy) was applied at 4 hours later. Treatments were also conducted on Day 3 and Day 6. The tumor, spleen, and tumor-draining lymph node (TDLN) were harvested for immune response profiling. Tumor tissues were cut into smaller pieces and digested with a mixture of DMEM supplemented with 1 mg/mL collagenase type V (Sigma, C6885), 1x GlutaMax (Gibco, 35050061), and 100 U/mL DNase I (Invitrogen, 18047019) at 37 °C for 45 minutes. The digested tissue was then meshed through a 250 μm tissue strainer (Pierce, 87791). The single-cell suspensions were washed with cold sterile PBS and resuspended in the staining buffer. After counting and aliquoting, cells were stained with fluorophore-conjugated antibodies for 30 minutes at 4 °C. The spleens and lymph nodes were processed following similar procedures, except a 70 μm cell strainer (Corning Falcon, 352235) was used and collagenase type V was omitted. Collected cells were pre-treated with CD16/32 antibody (Biolegend, 101320; 1:1000 dilution) to block Fc receptor-mediated nonspecific binding. After blocking, cells were stained with target-specific antibodies for 30 minutes, fixed with IC fixation buffer for 20 minutes and permeabilized using permeabilization buffer (Invitrogen, 00833356) to stain intracellular markers. The stained cells were analyzed by flow cytometry using an Agilent NovoCyte Quanteon, with compensation performed using the AbC total antibody compensation bead kit (Invitrogen, A10497). Fluorescence-labeled antibodies used in this study included CD8 (Biolegend, 100751), MHCII (Biolegend, 107639), CD3 (Biolegend, 100210), CD103 (eBioscience™, 46-1031-82), CD11c (Biolegend, 117308), IFN-γ (Biolegend, 505810), CD11b (Biolegend, 101226).

Therapeutic efficacy. 1×10^6 MOC1 or MOC2 cells in 50 μL PBS were subcutaneously injected into the right flank of 6 weeks-old female C57BL/6 mice to establish a head and neck tumor model (n = 5 for each group). When tumors reached 50 mm^3, FA-SCNPs (3.25 mg in 30 μL PBS) was injected intratumorally every three days for a total of three doses. For combination treatment, 5 Gy irradiation was applied 4 hours after FA-SCNP administration, and α-CD8 antibody (BioXCell, BE0061, 10 mg/kg) was administered intraperitoneally three times weekly for 2 weeks, initiating one day after first dose of FA-SCNP treatment. Tumor growth and body weight were monitored and recorded every other day. Tumor volumes were calculated as [length × $width^2$ /2]. At the experiment endpoint, tumors and major organs (heart, liver, spleen, lung, and kidney) were collected for histological examination including H&E and Ki-67 immunostaining.

In vivo fluorescence imaging. Fluorescence imaging was performed on an IVIS SpectrumCT 2 in vivo imaging system (Revvity). Cy5.5 labeled FA-SCNPs were administered intratumorally (i.t.) into MOC1 tumor-bearing mice (n = 3) when tumors reached approximately 100 mm^3. Mice were anesthetized with 2.5% isoflurane in oxygen during imaging. Images were acquired at 1, 4, and 24 hours post-injection using the Cy5.5 filter set (excitation: 675 nm; emission: 720 nm). For the *ex vivo* distribution study, mice were sacrificed 24 h post-injection. The main organs were collected for IVIS observation including the heart, liver, spleen, lung, kidney and tumor. Signal quantification was performed by drawing regions of interest (ROIs) around the excised organs using Living Image® 4.8.2 software (Revvity). The values represent the average radiant efficiency [p/s/cm^2/sr]/[μW/cm^2].

Statistical analysis: For *in vitro* study, all experiments were performed in triplicate. For *in vivo* study, growth curves are represented as mean ± SEM. All other data are represented as mean ± S.D. unless otherwise specified. One way ANOVA test was used to determine the statistical significance between groups with p <0.05 considered significantly different. *, $p < 0.05$; **, $p < 0.01$; ***, $p < 0.001$; ****, $p < 0.0001$; *ns*, no significant difference. All statistical analyses were performed with GraphPad Prism 9 software.

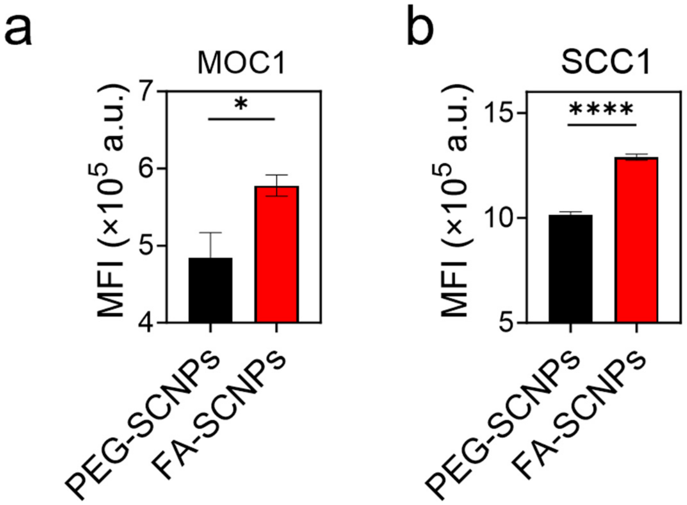

Figure S1. Cellular uptake of SCNPs (Rhodamine B-labeled) in different cell lines. (a) Mean fluorescence intensities (MFI) of the Rhodamine B signal in MOC1 cells. (b) MFI of the Rhodamine B signal in SCC1 cells. Statistical difference was evaluated by unpaired t test. *, $p < 0.05$; ****, $p < 0.0001$

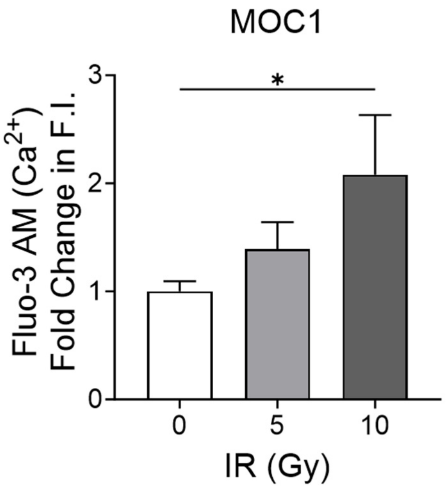

Figure S2. Changes in intracellular calcium levels in MOC1 cells treated with increasing doses of irradiation (IR), measured using Fluo-3 AM. Fluorescence signals were normalized to those of untreated cells. Data are represented as mean ± SEM. Statistical difference was evaluated using a one-way ANOVA test. *, $p < 0.05$.

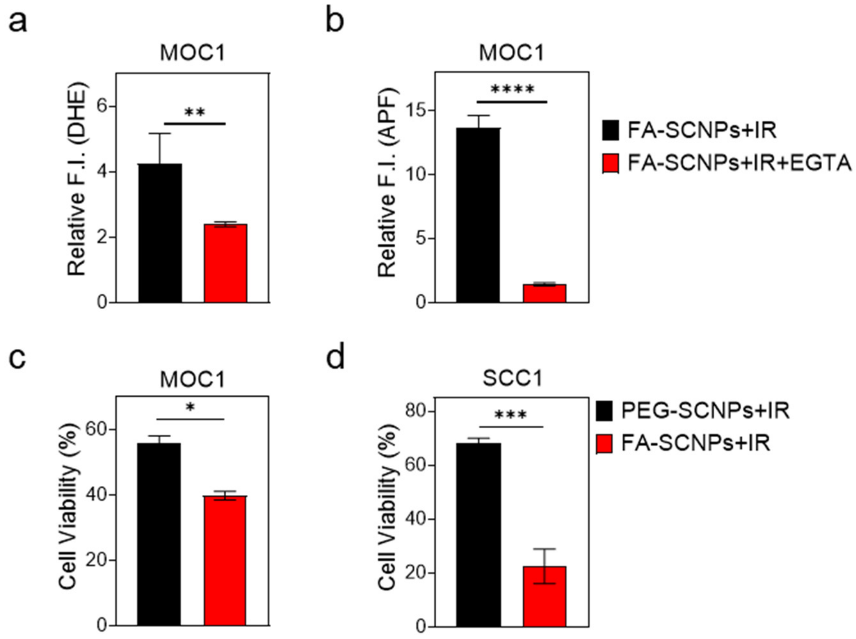

Figure S3. *In vitro* evaluation of the radiosensitizing effects of FA-SCNPs. FA-SCNPs (25 µg/mL) were incubated with MOC1 cells with irradiation (IR, 5 Gy) performed 4 hours after the administration of FA-SCNPs. Assays were conducted at 24 hours post adding FA-SCNPs. (a, b) Cellular levels of (a) superoxide, measured using DHE; and (b) hydroxyl radicals, measured using APF. Relative fluorescence intensity (F.I.) was normalized to control (PBS group). (c, d) Cell viability of MOC1 and SCC1 cells. For comparison, PEG-SCNPs (25 µg/mL) were also tested. (c) in MOC1 cells. (d) in SCC1 cells. Data are represented as mean ± SD. Statistical difference was evaluated by unpaired t test. *, $p < 0.05$; **, $p < 0.01$; ***, $p < 0.001$; ****, $p < 0.0001$.

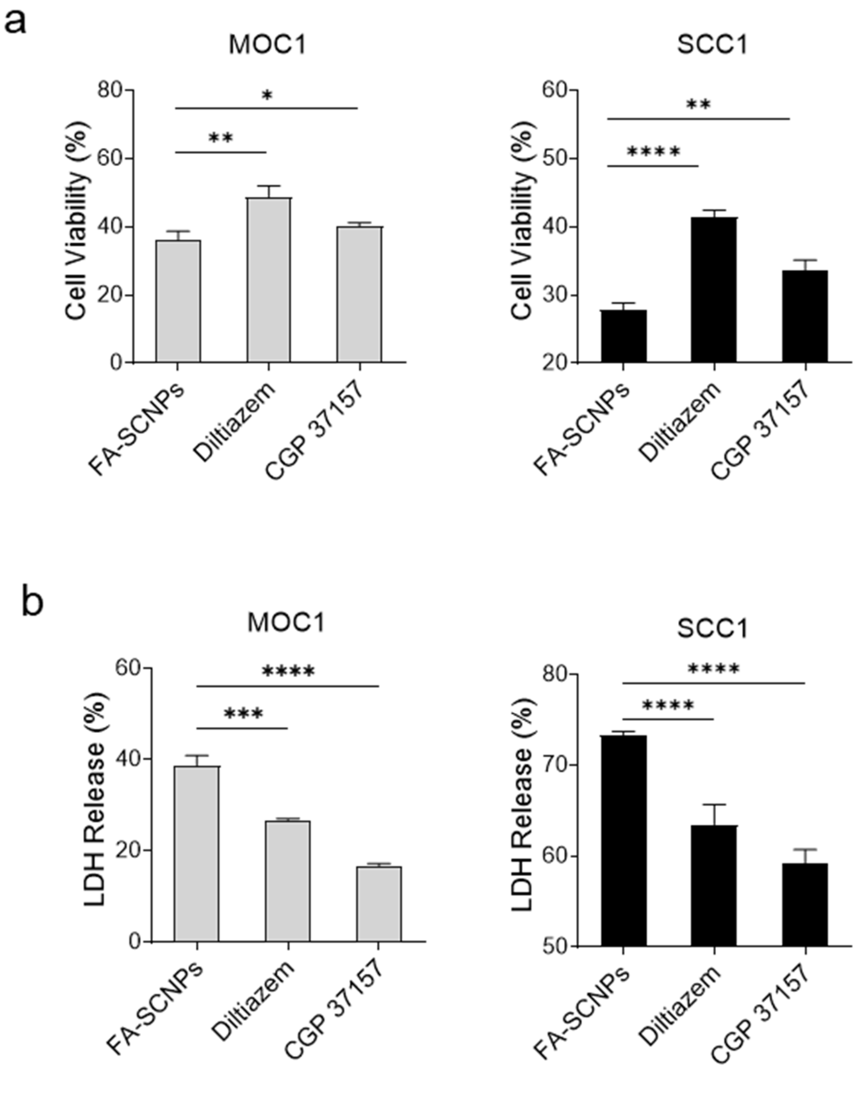

Figure S4. Cell viability and LDH release in MOC1 and SCC1 cells treated with FA-SCNPs in the presence of sodium-calcium exchanger (NCX) inhibitors. (a) Cell viability (b) LDH release. Data are represented as mean ± SD. Statistical difference was evaluated using a one-way ANOVA test. *, $p < 0.05$; **, $p < 0.01$; ***, $p < 0.001$; ****, $p < 0.0001$.

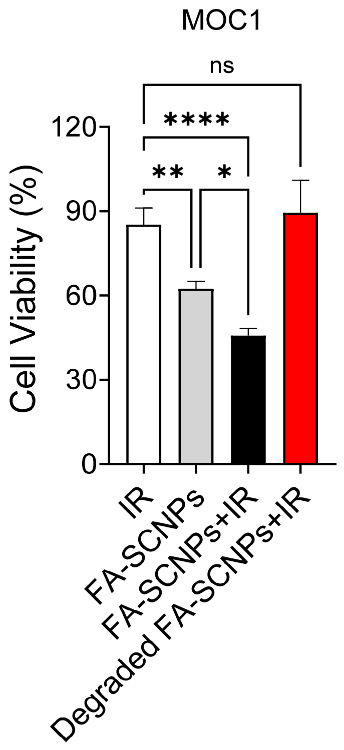

Figure S5. Comparison of the radiosensitizing effects of fresh versus aged FA-SCNPs. MOC1 cancer cells were treated with: (i) radiation only (5 Gy) (IR), (ii) FA-SCNPs alone, (iii) FA-SCNPs (25 µg/mL) + IR (5 Gy), or (iv) degraded FA-SCNPs (aged in water for 1 week prior to experiments, 25 µg/mL) + IR (5 Gy). Cell viability was measured 24 h post-treatment (n = 5). Data are presented as mean ± SD. Statistical significance was determined using a one-way ANOVA. *, $p < 0.05$; **, $p < 0.01$; ****, $p < 0.0001$; *ns*, not significant.

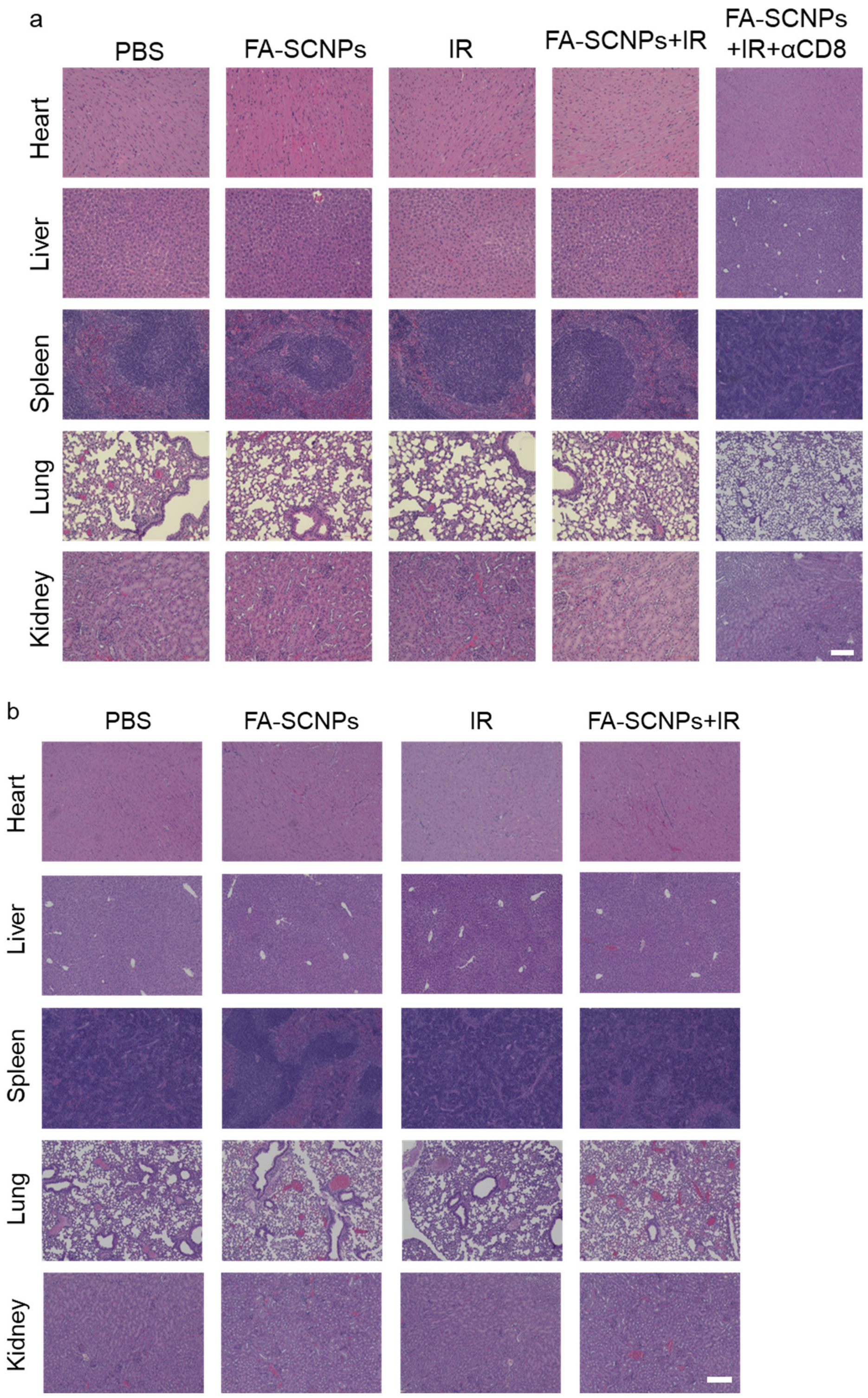

Figure S6. H&E staining of major organs from studies detailed in Figure 5. (a) Results from studies performed in MOC1 tumor-bearing mice. (b) Results from studied performed in MOC2 tumor-bearing mice. Scale bar: 20 μm.

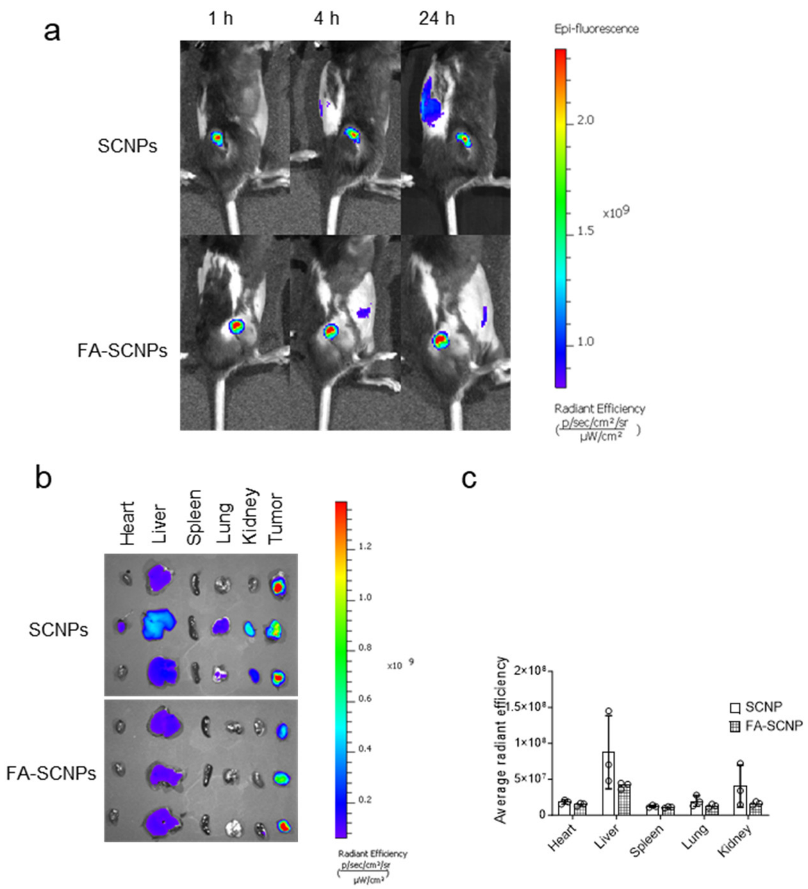

Figure S7. Distribution of SCNPs after intratumoral (i.t.) injection. Cy5.5-labeled SCNPs and FA-SCNPs were administered intratumorally (n = 3). (a) Whole-body fluorescence imaging at 1, 4, and 24 hours. (b) *Ex vivo* imaging of tumors and major organs harvested after the 24-hour imaging. (c) Histogram showing biodistribution based on the *ex vivo* imaging results.

REFERENCE

[1] Song, S.; Liu, D.; Peng, J.; Deng, H.; Guo, Y.; Xu, L. X.; Miller, A. D.; Xu, Y. Novel peptide ligand directs liposomes toward EGF-R high-expressing cancer cells in vitro and in vivo. *FASEB J* **2009**, *23* (5), 1396–1404.
[2] Ikeogu, N. M.; Edechi, C. A.; Akaluka, G. N.; Feiz-Barazandeh, A.; Uzonna, J. E. Isolation and preparation of bone marrow-derived immune cells for metabolic analysis. *Immunometabolism: Methods and Protocols* **2020**, 273–280.
